# Bronchial systemic-artery-to-pulmonary-artery-fistula as mimicker of pulmonary embolism: computed tomography findings verified with bronchial artery angiography

**DOI:** 10.1093/bjrcr/uaaf027

**Published:** 2025-05-05

**Authors:** Gernot Rott

**Affiliations:** Department of Radiology, Bethesda Hospital, Duisburg 47053, Germany

**Keywords:** pulmonary embolism, mimicker, systemic-artery-to-pulmonary-artery fistula, CT pulmonary angiography, bronchial angiography

## Abstract

We present the case of an 81-year-old patient, who was transferred to our department for diagnostic work-up and treatment of hemoptysis of the right lung. Two-phase contrast-enhanced chest CT revealed filling defects in right upper lobe and middle lobe pulmonary artery during the pulmonary-artery phase who vanished in the subsequent aortographic phase, consistent with systemic-artery-to-pulmonary-artery fistulas (SA-PAFs) of right bronchial artery mimicking pulmonary embolism. Selective bronchial catheter-arteriography confirmed bronchial SA-PAFs and bronchial artery embolization was performed effectively and without complication. For the best of our knowledge, this is the first case of a bronchial systemic-artery-to-pulmonary-artery-fistula mimicking PE directly correlated and proved with bronchial artery angiography.

## Introduction

Not every hypodense filling defect of a pulmonary artery depicted at CT angiography is a pulmonary embolism (PE). Tumours, such as pulmonary artery sarcoma, streak artefacts and in particular systemic-artery-to-pulmonary-artery fistulas (SA-PAFs) are well-known conditions, that can be misinterpreted as pulmonary embolism. On the other hand, pulmonary embolism is not a frequent, but at least an uncommon cause for hemoptysis^1^ and patients with hemoptysis often are referred to CT with question of or to rule out PE. This connection can lead to relevant misdiagnosis, as hemoptysis frequently is associated with bronchial systemic-artery-to-pulmonary-artery-fistula (B-SA-PAF).^2^

## Case presentation

An 81-year-old male patient was referred to our institution by a neighbouring hospital for endovascular treatment of severe hemoptysis of the right upper lobe caused by acute exacerbation of chronic bronchitis. He had a past medical history of recurrent pleural empyema, state after pleural drainage and decortication and CT findings of chronic cavitating pneumonia of the right lung. In the referring hospital bronchoscopy with clot extraction and endobronchial epinephrine injection of the right upper lobe had been performed. Under ongoing antibiotic therapy relevant laboratory parameters were within normal limits, except of a slightly elevated C-reactive protein (0.7 mg/L) and slightly lowered haemoglobin level (12.5 gm/dL).

**Figure 1.**
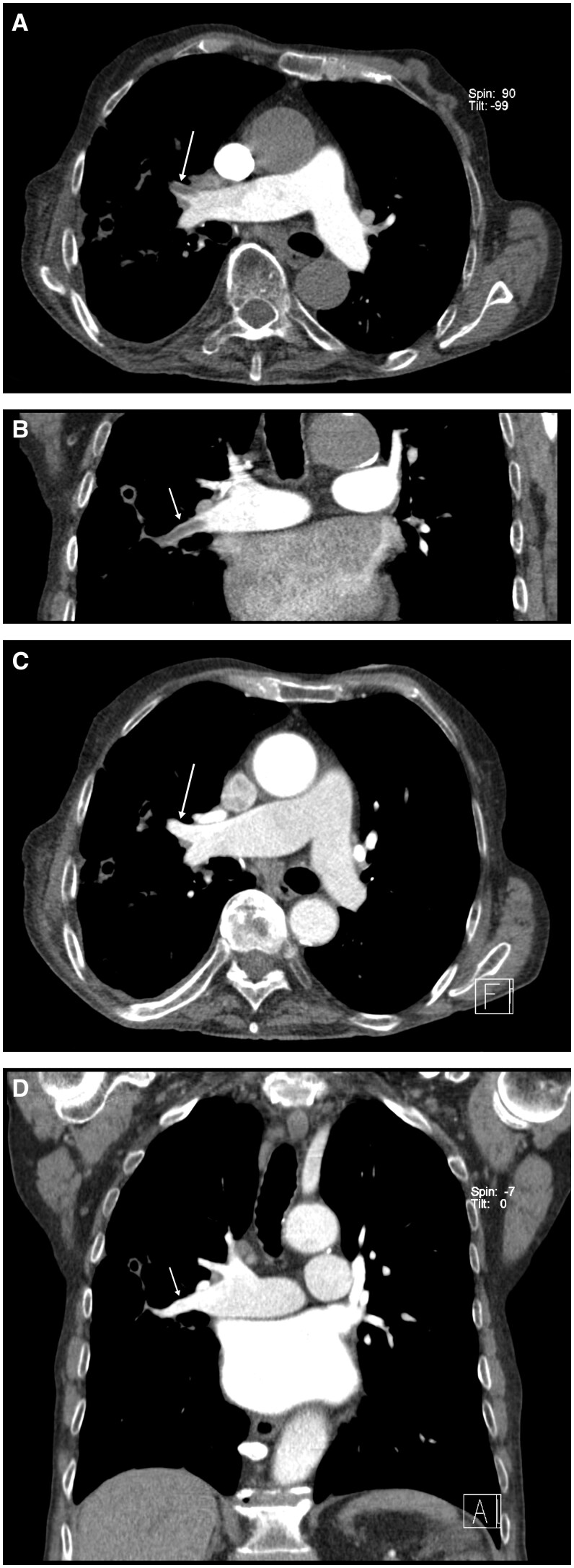
Contrast-enhanced CT. (A) Axial oblique plane: pulmonary phase with filling defects of middle lobe artery (arrow) consistent with pulmonary embolism (seemingly incomplete enhancement of the left posterior segmental artery due to partial volume effect). (B) Same situation in coronal oblique plane (arrow). (C) Axial oblique plane: aortographic phase with disappearance of filling defect (arrow). (D) Same in coronal oblique plane (arrow).

**Figure 2.**
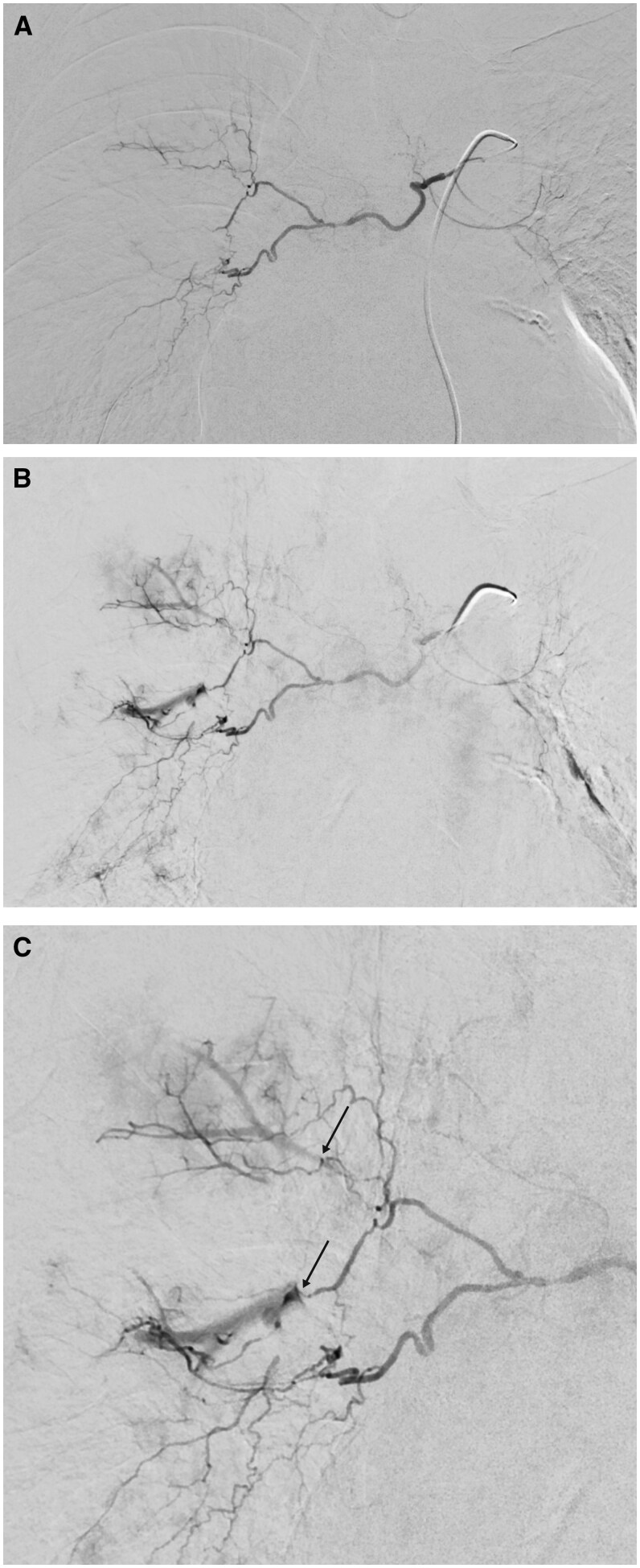
Arteriogram of right bronchial artery. Normal-sized right bronchial artery with common trunk with small left bronchial artery. (A) Early phase with no irregularities. (B) A few seconds later. Bronchial-to-pulmonary-artery fistula of right upper-lobe and middle-lobe artery. (C) Enlarged view (arrows indicate fistulas).

We carried out an actual two-phase contrast-enhanced chest CT. Pulmonary phase showed longitudinal filling-defects of pulmonary arteries of the right upper and middle lobe, which however had disappeared in the aortographic phase (Figure 1). Beyond this, no pathologic vessels were seen, bronchial arteries were not enlarged, no aberrant bronchial artery or non-bronchial SA-PAF^3^ was detectable. Diagnosis of SA-PAFs of the right bronchial artery was made. Catheter-angiography of bronchial arteries from a right femoral approach confirmed the diagnosis of B-SA-PAFs of right upper and middle lobe (Figure 2) and embolization of the right bronchial artery with *n*-butyl-2-cyanoacrylate (NBCA; Histoacryl; B. Braun, Melsungen, Germany) mixed with iodized oil (Lipiodol Ultra-Fluid; Guerbet, Roissy, France) at a ratio of 1:3 was performed without complication. Patient was sent back promptly to the neighbouring hospital, where he was discharged three days later without recurrence of hemoptysis.

## Discussion

For one thing, CT pulmonary angiography (CTPA) is standard technique for evaluation of PE. Similarly, in general, all patients with severe hemoptysis are examined with chest CT, sometimes with the explicit question of or to rule out PE. Here, intraluminal filling defects of pulmonary arteries are considered diagnostic of pulmonary thromboembolism.

Then again, patients with chronic inflammatory or neoplastic lesions of lungs and patients with chronic thromboembolic pulmonary hypertension frequently have compromised pulmonary artery circulation in affected regions of the lung. In these areas, the bronchial arteries can be recruited for circulation and pre-existing anastomosis between bronchial arteries and pulmonary vessels can re-open or re-develop. By this means, a bronchial artery to pulmonary artery shunt, anastomosis, or better called fistula may be created.

Bronchial artery to pulmonary artery fistula is the most frequent form of SA-PAF^4^ and often recognized during bronchial angiography performed for diagnosis and treatment of hemoptysis.^2^

However, SA-PAF, whether bronchial or transpleural/non-bronchial,^3^ is a well-known mimicker of acute PE.^4–9^ In a single-phase contrast-enhanced CT of the chest with pure pulmonary phase (CTPA) the not yet opacified blood at high pressure of the bronchial artery enters the contrast-enhanced blood of low pressure of the pulmonary artery in a mostly antegrade jet-like manner producing a longish shape hypodense filling defect in the pulmonary artery and can lead to misdiagnosis of SA-PAF as PE.^8^

PE is relatively rare among patients presenting with hemoptysis^1^ and hemoptysis and PE are two emergencies requiring completely different treatments. Consequence of misdiagnosis of B-SA-PAF as PE could be anticoagulation, which may lead to devastating worsening in patients with hemoptysis.^10^

## Learning points

- A bronchial systemic-artery-to-pulmonary-artery fistula can mimic pulmonary embolism in a single-phase CT angiography with pure pulmonary phase (CTPA).
- To avoid this misdiagnosis CT of the chest should not be performed only in a pure pulmonary phase, but additionally in an aortic phase or better in one combined pulmonary-aortic phase, where all pulmonary and systemic arteries are well opacified.
